# Reliable Out-of-Distribution Recognition of Synthetic Images

**DOI:** 10.3390/jimaging10050110

**Published:** 2024-05-01

**Authors:** Anatol Maier, Christian Riess

**Affiliations:** Department of Computer Science, IT Security Infrastructures Lab, University Erlangen-Nürnberg (FAU), 91058 Erlangen, Germany

**Keywords:** synthetic image detection, out-of-distribution examples, Bayesian Neural Networks, variational inference

## Abstract

Generative adversarial networks (GANs) and diffusion models (DMs) have revolutionized the creation of synthetically generated but realistic-looking images. Distinguishing such generated images from real camera captures is one of the key tasks in current multimedia forensics research. One particular challenge is the generalization to unseen generators or post-processing. This can be viewed as an issue of handling out-of-distribution inputs. Forensic detectors can be hardened by the extensive augmentation of the training data or specifically tailored networks. Nevertheless, such precautions only manage but do not remove the risk of prediction failures on inputs that look reasonable to an analyst but in fact are out of the training distribution of the network. With this work, we aim to close this gap with a Bayesian Neural Network (BNN) that provides an additional uncertainty measure to warn an analyst of difficult decisions. More specifically, the BNN learns the task at hand and also detects potential confusion between post-processing and image generator artifacts. Our experiments show that the BNN achieves on-par performance with the state-of-the-art detectors while producing more reliable predictions on out-of-distribution examples.

## 1. Introduction

Generative adversarial neural networks (GANs) [1] and diffusion-based neural networks (DMs) [2,3] pushed the door wide open regarding widely available, easy-to-use, and high-quality synthetic image generation and editing. This new technology is a powerful tool for any type of creative user [4,5,6]. On the downside, this advancement also opened the door to potentially malicious exploitation, oftentimes summarized as the threat of so-called DeepFakes. Hence, from a forensic perspective, it is important to research robust and reliable techniques for the detection of synthetically generated media content.

The detection of generated content can be performed at different levels of abstraction. Generators may introduce semantic issues involving inconsistencies in lighting [7,8] or the eyes of people, etc. [9,10]. However, one may expect that such artifacts will be gradually removed as generators progress towards modelling increasing amounts of contextual knowledge. Lower-level statistical traces can provide alternative cues for detecting generated content. For example, it has been shown that GANs and DMs exhibit artificial statistical fingerprints that can form the basis not only for distinguishing real from artificial images but also for the attribution of synthetic images to their generator network [11,12,13].

The detectors for such forensic cues are typically neural networks. Such learning-based systems implicitly assume that the training data are representative of the test inputs in the field. If a test input differs too much from the training distribution, then the output of the network is undefined. In such cases, neural networks have a tendency to perform erroneous predictions with high confidence [14]. This issue is known as the training–test mismatch.

The sensitivity of learning-based methods to training–test mismatches poses a severe challenge for multimedia forensics in general and the task of unconstrained synthetic image detection in particular. Since blind multimedia forensics is by definition concerned with samples from unknown origins, forensic methods need to take care of mitigating the training–test mismatch. Such mismatches are difficult to avoid due to limits regarding the information about the structure, the mode of operation, and the underlying training of an image generation model just from an image itself during testing. Additionally, the potential post-processing on the distribution channel might be unknown. While extensive data augmentation helps to reduce the gap between training and test data [15,16,17], it is virtually impossible to consider all the potential GAN and DM architectures and post-processing operations in the training. However, even slight mismatches between training and test data can lead to failures [18,19,20].

To alleviate this issue, this work aims to provide additional tools for the reliable detection of synthetically generated images. We investigate the suitability of a Bayesian Neural Network (BNN) as a learning-based detector with a built-in uncertainty measure. If the network is evaluated on inputs for which it has not properly been trained, then the result exhibits higher uncertainty. That way, an analyst can detect cases where she cannot trust the outcome of the classifier. Moreover, we train the network with dedicated predictors for JPEG compression and resampling, which are highly common operations in social media and simultaneously notorious sources of out-of-distribution data. The feature activation similarity of the predictions of these dedicated output nodes further aids in exhibiting the out-of-distribution samples. Our experiments show that the detection performance of the network is comparable to related work, but we show that its added benefit of the uncertainty measure can benefit the practical use by avoiding false decisions. In summary, our main contributions are the following:

- We propose a Bayesian Neural Network (BNN) for synthetic image detection, with the particular benefit to detect out-of-distribution inputs.
- The network architecture particularly benefits from a multi-class approach, with separate output nodes for real images, synthetic images, and JPEG compression.
- We additionally propose the feature activation similarity as an indicator of the failure cases in the out-of-distribution detection.

The remainder of this paper is organized as follows. Section 2 reviews the related work and discusses the limitations of traditional CNNs. Section 3 introduces the theory of variational inference-based Bayesian Neural Networks and noise contrastive estimates for classification. Section 4 details the experimental setup to assess the model’s performance within the in-distribution as well as out-of-distribution domains. Section 5 presents the experimental results, and Section 6 concludes this work.

## 2. Related Work

Early research that explored the detection of synthetically GAN-generated images took inspiration from the classical multimedia forensics task of camera device identification [21,22]. Marra et al. [11] show the existence of statistical GAN fingerprints similar to camera fingerprints. In a similar spirit, Yu et al. demonstrate that fake images generated by various traditional generation models can be attributed to their sources [12]. They reveal the fact that these traditional generation models leave fingerprints in the generated images. The authors also show that these fingerprints highly depend on the specific architecture and its parameterization. Further, Wang et al. show that a CNN model is able to distinguish between the real images and fake images generated by various types of GANs [15]. According to the authors, GAN-generated images share common defects, enabling their separation from real images. Girish et al. further propose a new attribution method to deal with the open-world scenario where the detector has no knowledge of the generation model [23]. Diffusion models are the more modern replacement of GANs. Here, Sha et al. investigate the possibility of distinguishing real images from the images generated by text-to-image models [24].

However, in these aforementioned works, robustness considerations such as the performance on unseen postprocessing typically play a secondary role or are not investigated at all. Corvi et al. highlight the importance of robustness [18]. In their work, it is shown that most detectors trained on GAN images are challenged to operate decently when confronted either with DM-based images or synthetic images that have undergone unseen JPEG compression. Probably the most widely used strategy regarding the robustness of detectors is data augmentation, which has been applied and investigated in various forensics works [15,16,17]. However, augmentation requires an enumeration of the expected influencing factors, which quickly leads to a combinatorial explosion of possibilities. This difficulty is exacerbated by the fact that small deviations between the training and test data may already deteriorate the classifier performance [18,19,20]. In contrast to data augmentation that aims to avoid unseen data at all, we propose to use a Bayesian Neural Network that produces as a byproduct an uncertainty measure. It is also very reasonable to augment the training data of that classifier, but its uncertainty-based design acknowledges the difficulty to fully anticipate all the data statistics that might occur in the field. An analyst can use that uncertainty measure to either abstain from a decision or to retrain the classifier for the specific use case.

There exist other forensic works that specifically focus on reliability. For example, Güera et al. and Salvi et al. explicitly model reliability as an embedding distance for images and speech [25,26]. Guillaro et al. provide a learned confidence for the case of image manipulation detection [27]. In contrast, the uncertainty measure of the proposed BNN is directly linked to the empirical variability of the outputs from an ensemble of classifiers and as such seeks to achieve trust from many consistent decisions.

In the broader field of machine learning research, the question of trust in model predictions has a long history [28,29]. One simple approach to uncertainty modelling is to interpret the maximum output of a neural network with softmax activation as the confidence associated with the prediction. However, standard neural networks perform poorly at quantifying predictive uncertainty, providing misleading and overconfident confidence estimates [14,30]. Nevertheless, Hendrycks and Gimpel observe that the prediction probability for out-of-distribution examples tends to be lower than for in-distribution examples, thereby providing a baseline regarding the detection of abnormal examples based on softmax statistics [30].

In some cases, missing or biased confidence estimates can be added or rescaled post hoc by calibration to the true accuracy. Previous work on post hoc calibration addresses, for example, support vector machines [31], boosted trees [32], and deep neural networks [33].

Recent efforts have focused on combining neural networks with Bayesian methods as a principled way to reason regarding predictive uncertainty. In a Bayesian Neural Network, each parameter is represented by a probability distribution that captures the uncertainty regarding its value. Training a BNN involves obtaining the posterior distribution over the parameters. The analytic integration over the whole parameter space of a neural network is intractable, and practical methods resort to either approximation or simulation techniques. To this end, the recent developments on variational Bayes have led to the increasing popularity of stochastic variational inference (SVI) [34,35,36]. SVI has recently also been examined on large datasets such as CIFAR-100 [37]. In this work, we investigate such a BNN that is trained with SVI.

## 3. Bayesian Neural Networks for Reliable Synthetic Image Detection

This section introduces two techniques that are central for classification under uncertainty. First, we describe the BNN with stochastic variational inference for quantifying uncertainty. Second, we describe noise contrastive priors for improved representation of the uncertainty.

### 3.1. Variational Inference

Traditional neural networks maximize the posterior distribution during training. In contrast, the Bayesian formulation seeks to find the posterior distribution itself. Unfortunately, it is intractable to find that posterior distribution analytically since that would require integration over the space of all possible weight configurations of the network.

One tractable alternative is to use an approximate solution via stochastic variational inference (SVI) [36]. Here, the intractable posterior p(w|D) over the weights w after seeing the data D is approximated by a tractable variational posterior q(w|θ) with parameters θ. Variational inference then seeks optimal variational parameters $\theta^{*}$ that minimize the Kullback–Leibler (KL) divergence between the variational posterior q(w|θ) and the true unknown posterior distribution p(w|D), which is defined as

(1) $$\begin{array}{cc} \theta^{*} & =\underset{\theta }{\mathrm{argmin}}\mathrm{KL}\left[q(w|\theta )\;||\;p(w|D)\right]\\ & =\underset{\theta }{\mathrm{argmin}}\int q\left(w|\theta \right)\log\frac{q(w|\theta )}{p(w|D)}\;dw\\ & =\underset{\theta }{\mathrm{argmin}}\;\underset{\mathrm{variational}\;\mathrm{free}\;\mathrm{energy}\;F(D,\theta )}{\underbrace{\mathrm{KL}\left[q(w|\theta )\;||\;p(w)\right]-E_{q(w|\theta )}\left[\log p(D|w)\right]}}\;. \end{array}$$

Here, the variational free energy F(D,θ) is also referred to as the negative evidence lower bound (ELBO). F(D,θ) is the objective function that we seek to minimize in order to find the optimal parameters $\theta^{*}$. The variational free energy can be further decomposed into two components, namely the complexity cost and the likelihood cost,

(2) $$\begin{array}{cc} F(D,\theta ) & =\underset{\mathrm{complexity}\text{-}\mathrm{cost}}{\underbrace{\mathrm{KL}\left[q(w|\theta )\;||\;p(w)\right]}}-\underset{\mathrm{likelihood}\text{-}\mathrm{cost}}{\underbrace{E_{q(w|\theta )}\left[\log p(D|w)\right]}}\\ & =E_{q(w|\theta )}\log q\left(w|\theta \right)-E_{q(w|\theta )}\log p\left(w\right)-E_{q(w|\theta )}\log p(D\left|w\right)\\ & \approx \frac{1}{T_{\mathrm{train}}}\sum_{i=1}^{T_{\mathrm{train}}}\left[\log q(w^{\left(i\right)}\left|\theta \right)-\log p\left(w^{\left(i\right)}\right)-\log p\left(D|w^{\left(i\right)}\right)\right]\;, \end{array}$$

which can be approximated by drawing $T_{\mathrm{train}}$ times weights $w^{\left(i\right)}$ from q(w|θ). Solving the optimization problem as defined in Equation (2) yields the optimal parameters $\theta^{*}$.

Given then the variational distribution $q(w|\theta^{*})$, the predictive distribution is approximated as

(3) $$\begin{array}{cc} p(y^{*}\left|x^{*},D\right) & =\int p\left(y^{*}|x^{*},w\right)p(w\left|D\right)\;dw\\ & \approx \int p(y^{*}\left|x^{*},w\right)q(w\left|\theta^{*}\right)\;dw\;. \end{array}$$

The network prediction for an input is an estimator of the expectation, and the associated uncertainty is an estimator of the predictive variance. Both the expectation and the predictive variance are obtained via sampling from the variational posterior. Specifically, the expectation is provided by

(4) $$\begin{array}{cc} E_{q(w|D)}\left[p(y^{*}|x^{*})\right] & =\int p(y^{*}\left|x^{*},w\right)q\left(w|\theta^{*}\right)\;dw\\ & \approx \frac{1}{T}\sum_{t=1}^{T}P_{w^{\left(t\right)}}\left(y^{*}|x^{*}\right)\\ & =\bar{p}\left(y^{*}|x^{*}\right)\;, \end{array}$$

where $P_{w}$ denotes the neural network with a set of weights drawn from the variational posterior q(w|θ). Hence, the estimate for an unseen data point $x^{*}$ requires *T* draws and evaluations from the trained network. The unbiased predictive variance, which represents our model uncertainty, is then provided by the approximated expectation defined in Equation (4) and the definition of the variance

(5) $$\mathrm{Var}\left[p(y^{*}|x^{*})\right]=\frac{1}{T-1}\sum_{t=1}^{T}{\left(P_{w^{\left(t\right)}}(y^{*}\left|x^{*}\right)-\bar{p}\left(y^{*}|x^{*}\right)\right)}^{2}\;.$$

### 3.2. Noise Contrastive Prior Estimation

The noise contrastive estimation (NCE) is an augmentation technique where a model is contrasted with random noise during training. The objective is to discriminate training data from noise data sampled from an artificial noise distribution, which is considered out-of-distribution (OOD). Therefore, by employing NCE, a trained classifier can estimate the probability of a data sample belonging either to the training or to the noise distribution. This technique is therefore well-suited within a probabilistic model to obtain more reliable uncertainty estimates. In general, obtaining OOD data is not trivial. In practice, it is often sufficient to add noise to the training data to generate OOD samples near the boundary of the training data distribution. According to Hafner et al. [38], this approach also yields reliable uncertainty estimates in other regions of the OOD space, upon which the approach of noise contrastive priors (NCPs) is based.

In this work, we follow the derivation of Hafner et al. [38] to define NCP for functional uncertainty estimations. For classification, a noise contrastive prior forms a joint distribution p(x,y) over input *x* and class *y*, which can be rewritten as the product of an input prior p(x) and an output prior p(y|x).

We set the input prior as

(6) $$p_{nc}\left(\hat{x}\right)=\frac{1}{N}\sum_{i=1}^{N}N(\hat{x}-x_{i}\left|0,\sigma_{x}^{2}\right)\;,$$

where $x_{i}$ indicates the training data and $\hat{x}=x_{i}+\epsilon$ describes the distribution of OOD data corrupted by random noise $\epsilon ∼N(0,\sigma_{x}^{2})$ with hyperparameter $\sigma_{x}$.

The output prior is defined such that the model shall predict the correct target *y* for input *x* as well as for perturbed input $\hat{x}$. Within our categorical classification setting, the output prior is therefore defined as a Bernoulli distribution

(7) $$p_{nc}(\hat{y}\left|\hat{x}\right)=y^{k}\cdot {\left(1-y\right)}^{\left(1-k\right)},$$

where *k* is a hyperparameter that models the probability of success and should for OOD input result in high prior uncertainty. To generate an output at *x*, we first sample from the variational distribution q(w|θ) and then use that sample as input for Equation (7), from which we finally sample an output value *y*. The predictive uncertainty is then reflected by the variance over the output as defined in Equation (5). By minimizing the KL divergence between the variational posterior q(w|θ) and a prior over weights p(w), we encourage the model to express low uncertainty within in-distribution domains. Conversely, we can enforce high uncertainty in OOD regions through comparison to an NCP. Through parameterization of the KL divergence from the weight space into the output space, we can obtain a convergence between expected output, epistemic uncertainty, and the mean prior for OOD inputs. This is possible due to the variational distribution q(w|θ) inducing a distribution q(μ|x,θ) in data space. Therefore, by replacing q(w|θ) with $q(\mu \left|\tilde{x},\theta \right)$ and p(w) with $p_{nc}(\tilde{y}\left|\tilde{x}\right)$ and using an OOD dataset $\tilde{x},\tilde{y}$ derived from our training dataset x,y, the loss function then becomes

(8) $$L\left(\theta \right)\approx -E_{q(w|q)}\log p(y\left|x,w,\theta \right)+\mathrm{KL}\left(q(\mu \left|\tilde{x},\theta \right)\;||\;p_{nc}(\tilde{y}\left|\tilde{x}\right)\right).$$

Equation (8) yields an approximation of Equation (2) for reasons explained in Appendix B by Hafner et al. [38]. For their experiments, the authors use the opposite direction of the KL divergence without having found a significant difference. The concrete loss function the authors use is defined as follows

(9) $$L\left(\theta \right)=-E_{q(w|q)}\log p(y\left|x,w,\theta \right)+\mathrm{KL}\left(p_{nc}(\tilde{y}\left|\tilde{x}\right)\;||\;q(\mu \left|\tilde{x},\theta \right)\right),$$

which we also employ in our work. This allows an interpretation of the KL divergence as fitting the mean distribution to an empirical OOD distribution using data augmentation.

## 4. Experimental Setup

This section reports the experimental setup, the model architecture, the training procedure, and the data generation for training and evaluation.

### 4.1. Model Architecture

As investigated by Corvi et al. [13], even modern and sophisticated generative models still leave exploitable traces within the spatial as well as spectral domains. According to the authors, a detector should therefore explore both. In line with their findings, we employ a wavelet transform prior to the model input to exploit features within the spatial as well as frequency domains. The input to the neural network is then the wavelet approximation alongside the frequency parts within the spatial domain.

**Figure 1:**
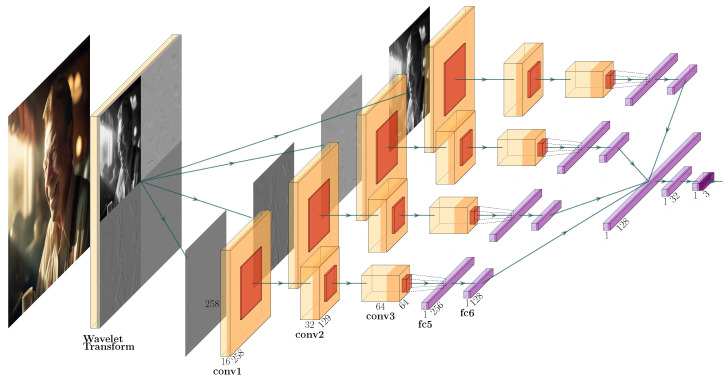
Architecture of the Bayesian Neural Network. The four wavelet sub-bands are used as separate inputs regarding a sequence of three convolutional layers followed by two fully connected layers used as a separate input, and target classes are “real”, “synthetic”, and “compressed”.

The proposed Bayesian Neural Network uses a convolutional architecture as a backbone. A visual representation of the proposed model is shown in Figure 1. As a first step, we apply a two-dimensional discrete wavelet transform with Daubechies 5 wavelets. This transforms the input into a joint spatio-frequency domain, losely following insights by Corvi et al. that generative models leave traces in the spatial and frequency domains [13]. Each of the four wavelet sub-bands is passed to a separate branch of the network. One branch consists of three convolutional blocks consisting of a 3×3 convolution followed by ReLU-activation and 2×2 max-pooling. The three convolutional blocks use an increasing number of kernels, namely 16, 32, and 64. Each third convolutional block is followed by two fully connected layers within the same branch. Then, the output of all four branches is concatenated and combined in two further fully connected layers. A final output layer distinguishes the three classes “real”, “synthetic”, and “compressed”. In the output layer, we use the Sigmoid activation function and therefore treat all three classes as non-exclusive. This way, the model is not forced to decide on a single class, and it could even completely abstain from a decision by assigning low scores to all output nodes.

For the initialization of the BNN’s variational posterior, we assume a normally distributed variational posterior. Hence, the BNN has approximately twice as many training parameters compared to a traditional CNN model due to the mean and standard deviation of each weight. The concrete implementation applies pseudo-independent weight perturbations based on the Flipout method [39]. Hence, to learn probability distributions over the weights, our implementation replaces the convolutional and fully connected layers with Flipout convolution and Flipout fully connected layers. The weight prior is a zero-mean Gaussian distribution with unit variance. The weights are initialized using the He normal weight initialization [40], as provided within the TensorFlow framework. During training, we draw 5 samples to calculate the predictive variance according to Equation (5) by randomly sampling weights from the BNN. For evaluation, we increase the sampling rate to 20 MC samples. For the comparative analysis in Section 5.2, we train our BNN model on ProGAN-generated data and therefore adjust the input layer resolution to 256×256 pixels. Within the further analysis, we use data generated by the stable diffusion model and adjust the input layer resolution to 512×512 pixels.

### 4.2. Training Parameters

The BNN model is trained with the Adam optimizer with a learning rate of $l=10^{-4}$, $\beta_{1}=0.9$, $\beta_{2}=0.999$, and $\epsilon =10^{-7}$. Furthermore, we use a batch size of 64. Each model is trained for a total of 30 epochs. The reported experimental results are based on the best-performing model in terms of validation loss during training, which is evaluated every epoch. For the training procedure of the BNN, we use the variational free energy loss from Equation (2) together with the NCP prior estimation as defined in Equation (8).

### 4.3. Datasets for Training and Generation of Training Data

For the evaluation in Section 5.2, we train our BNN model on the dataset by Wang et al. [15]. The dataset contains 363,000 real images from the LSUN dataset [41] and 362,000 images generated by 20 different ProGAN [42] models, each trained on a different LSUN object category. The 20 models arise from the fact that ProGAN images are limited to the specific image domain on which they are trained. All images have a resolution of 256×256 pixels. For model validation, we use a subset of 3200 images.

In Section 5.3 and later, we explore the influence of diffusion-based models, and we investigate the reliability on uncertainty estimates and possible confusion of generator artifacts and compression artifacts. To this end, we use the BNN trained on synthetic images from stable diffusion [43]. with image descriptions from the COCO dataset [44]. For real data, we use images from the COCO dataset [44]. Here, the training set consists of 118,000 synthetic and 118,000 real images. During training, we apply JPEG compression with probability $P_{jpeg}=0.7$ with a random quality factor between 65 and 100 using the TensorFlow built-in JPEG compression. During evaluation, JPEG images are compressed with Python Pillow version 9.3.0 and ImageIO version 2.31.4.

## 5. Experimental Results

We first examine the in-distribution detection performance for the synthetically generated images, followed by a comparison with the related work on various out-of-distribution cases. We then show how the uncertainty measure helps to provide reliable model predictions. Last, we explore a further possibility to recognize potential failure cases by cross-checking the results of the output nodes of the network.

### 5.1. In-Distribution Detection Performance

The in-distribution performance of the BNN is evaluated on a test dataset of additional images, namely 1600 real and 1600 synthetic images that were unseen during training but from the same data sources. These images are randomly JPEG-compressed analogously to the JPEG augmentation during training.

The evaluation results are shown in Table 1. The BNN performs almost perfectly well on all three tasks, namely the detection of real, synthetic, and compressed images. Overall, the BNN achieves an average F1-score of 0.970 and an average AU-ROC score of 0.993, which demonstrates that the BNN effectively learns the tasks at hand.

### 5.2. Out-of-Distribution Detection Performance and Comparison to Related Work

Our primary emphasis is on the generalization ability regarding data from generators that were unseen during training. Hence, this experiment shows the generalization performance of the BNN to various generative models on which it was not trained. Recall from Section 4.3 that the BNN is trained on synthetic ProGAN images and real images from the LSUN dataset. The testing is performed on a separate test dataset that was not observed during training. It comprises synthetic images from StyleGAN2 [45], StyleGAN3 [46], BigGAN [47], Dall-E mini [48], Dall-E 2 [2], stable diffusion [43], latent diffusion [3], and taming transformer [49]. For text-to-image generator models, we utilized the image descriptions provided by the COCO-datset [44]. The real data for testing are also from datasets that were unseen during training, namely COCO [44], ImageNet [50], and UCID [51].

The performance is compared to four related works for synthetic image detection, which are briefly introduced in this paragraph. Spec is a traditional approach based on frequency analysis [52]. PatchForensics analyzes the local image patches [53]. Wang et al. propose a learning-based approach using a ResNet50 architecture alongside post-processing augmentation [15]. Gragnaniello et al. refine the approach by Wang et al. by abstaining from downsampling within the first layer and introducing additional augmentation [15,19]. The results are reported using balanced accuracy and its associated area under the receiver-operating curve (AUC). For the comparative methods, we use the numbers as provided by Corvi et al. [18]. To ensure a fair comparison, we carefully follow the same evaluation protocol as Corvi et al. The only notable difference is that our testing data are smaller by a factor of 2; hence, we use 500 synthetic images from each generative model and 2500 real images.

Table 2 shows the results for the uncompressed synthetic images. Here, the detection only has to cope with the fact that the images come from unseen sources and generators, but no further post-processing is applied. The first row shows the detection performance on the in-distribution test set for the ProGAN images, and the following rows depict the performance on the out-of-distribution data. The last row shows the average performance of each method. The three rightmost columns depict the results of the BNN. Out of those three columns, the leftmost shows the BNN’s performance by selecting the most likely class. The middle column reflects the performance with an activation-based abstain threshold, where no decision is made if all the class activations are below a threshold of 0.5. The abstains opt out of the evaluation; i.e., the reported performance only includes the samples from which the BNN did not abstain. The rightmost column shows the performance with an uncertainty-based abstain threshold. Here, each sample with high uncertainty is flagged as unreliable and analogously abstains from prediction. The uncertainty threshold $\sigma_{abstain}=2\cdot \sigma_{in}=0.182$ is set based on the mean uncertainty regarding the in-distribution test set $\sigma_{in}=0.091$. A prediction is considered unreliable if the uncertainty exceeds the average in-distribution uncertainty $\sigma_{in}$ by a factor of two.

The results show that the performance of the BNN is comparable to related works on in-distribution data. The performance of the BNN also decreases on out-of-distribution data (as expected), with particularly weak spots on the StyleGAN3 and Dall-E 2 images. However, the ability to abstain from the decision can increase the performance across all the architectures.

Table 3 shows an analysis of the resized and compressed synthetic images, which is a more realistic and challenging scenario. Again, for a fair comparison, we follow the same post-processing approach of image resizing and additional JPEG compression as described by Corvi et al. [18]. The overall structure of the results is the same as in Table 2. In this more challenging scenario, Spec, PatchForensics, and Wang et al. drop to random guessing for all the generators [15,52,53]. Meanwhile, Gragnaniello et al. is able to retain decent performance for the other GAN-based generators, and it also drops to random guessing for the diffusion-based models [19]. The BNN also takes a performance penalty. However, it is able to retain decent performance for most of the GAN-based generators and for most of the diffusion-based generators, which again is slightly improved by utilizing our abstain policies. While the BNN shows on average on-par but slightly inferior performance regarding uncompressed data compared to Gragnaniello et al., it demonstrates higher robustness and stability within the more challenging setting [19].

### 5.3. Out-of-Distribution Detection via Uncertainty Estimates

This experiment analyzes the BNN-specific possibility to express uncertainty for the detection of out-of-distribution samples and for avoiding unreliable predictions.

The BNN’s uncertainty estimates are compared to the activation statistics as expressed by the traditional neural network models. Therefore, we additionally train a CNN model analogously to the BNN described in Section 4. Both models are evaluated on an out-of-distribution test set from various generators. More specifically, we include unseen in-distribution images from stable diffusion and out-of-distribution images from StyleGAN2 [45], Dall-E 2 [2], GLIDE [54], denoising diffusion probabilistic models (DDPM) [55], and the noise conditional score network (NCSNPP) [56]. Additionally, we include images from other real datasets unseen during training, namely the LSUN dataset [41] and the unconstrained face detection dataset (UFDD) [57].

For the BNN, we use the uncertainty estimates based on M=20 Monte Carlo draws for discrimination between the in-distribution and out-of-distribution samples. For the CNN, we interpret 1—class activation as a means of uncertainty. The results are reported in terms of the area under the receiver-operating curve (AUC).

**Figure 2:**
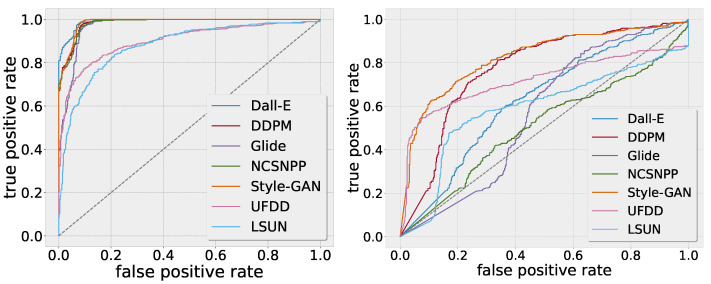
Detection of out-of-distribution examples of the BNN and CNN on the UFDD and LSUN datasets. Left: ROC curves of the BNN model. Right: ROC curves of the CNN.

Figure 2 shows the results, with an ROC curve for the BNN uncertainties on the left and an ROC curve for the CNN class activation uncertainties on the right. The uncertainty-based thresholding achieves decent results for all the unseen generative models as well as for the unseen real images. In contrast, the CNN class activations are considerably weaker indicators as to whether a sample is from the out-of-distribution domain.

### 5.4. Reliability Evaluation via Compression Similarity

**Figure 3:**
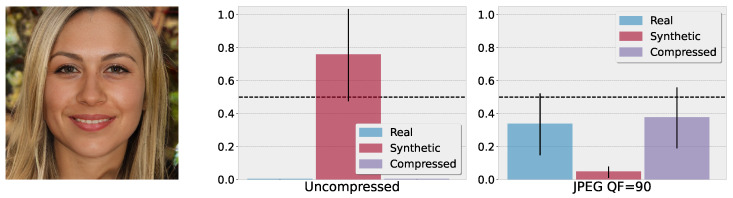
(**Left**) image generated by the EG3D model (out-of-distribution). (**Middle**) class activations for the uncompressed image. The BNN correctly shows a high activation for the synthetic class and a high uncertainty. (**Right**) class activations for the images after JPEG compression with quality factor Q=90. Here, the model becomes highly uncertain about its decision and abstains from a prediction. However, it can be observed that now the most likely classes are real alongside compressed.

The three output nodes, real, synthetic, and compressed, provide another angle for assessing the reliability of the predictions. Figure 3 shows a qualitative example that is generated by the EG3D model. The data from this model are not used during training. The middle plot shows the BNN’s class activation for the uncompressed version of this image, averaged over M=20 Monte Carlo draws. In this case, the BNN correctly shows a high activation for the synthetic class with $P_{synth}=0.78$ together with a high uncertainty of $\sigma_{synth}=0.32$. The right plot of Figure 3 shows the BNN’s class activation after compressing the image with a JPEG quality factor of Q=90. The prediction notably changes. The most likely predicted classes are now “real” alongside “compressed”, which would be a false decision. However, the BNN’s prediction is highly uncertain and the model abstains from a prediction as the mean activation for each class is below the threshold of 0.5, as indicated by the dotted line. The inability to reliably operate on that input is therefore reflected by the abstain decision, i.e., to not decide on any class together with the high uncertainties regarding the classes.

**Figure 4:**
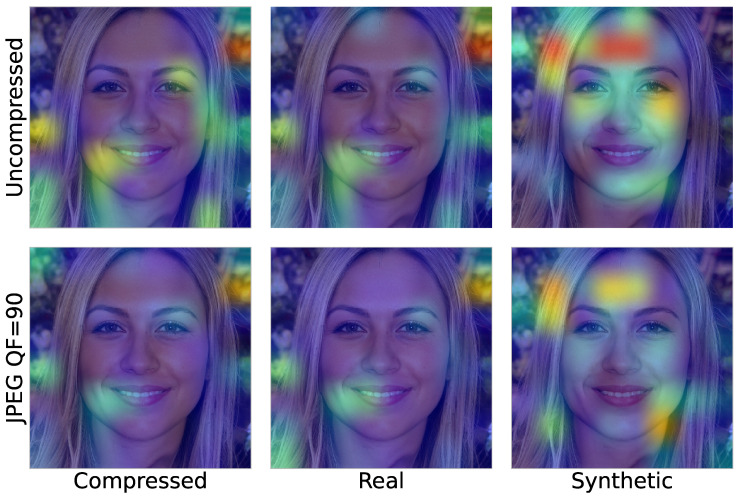
Activation heatmap of the BNN for a sample image from the EG3D model. Each column shows the activation for the corresponding class. In the top row are shown the respective activations in the uncompressed case. Here, for each class, different image regions are dominant, with some overlap between the real and compressed classes. The bottom row shows the respective class activations for the JPEG-compressed case. Here, the activation for the synthetic class becomes less dominant. Additionally, the activations for the compressed and real class share mostly the same regions, which is a telltale sign of unreliable post-processing confusion.

Another telltale sign that the decision is unreliable can be found when examining the image regions that are relevant for the BNN decision as produced by Grad-CAM [58] from the mean feature activation over M=20 Monte Carlo draws. Figure 4 shows the feature activations for each class that led to the respective decision from Figure 3. The top row shows the feature activation for the uncompressed image per output class. For each class, there are different regions in the image that are relevant, with a slight overlap between the “real” and the “compressed” class. The bottom row shows the feature activation for the JPEG-compressed image. Here, the feature activation for the “synthetic” class is weaker. Additionally, the relevant regions for the “compressed” and “real” classes are very similar, which is a telltale sign in terms of the unreliable confusion induced by the post-processing.

To quantify this property, we evaluate the error rate of the BNN for various in-distribution and out-of-distribution generators and datasets. For each dataset, we analyze 500 images and use M=20 Monte Carlo samples. Table 4 shows a quantitative analysis regarding the effectivity of the previously introduced activation-based abstain, uncertainy-based abstain, and the now-presented SSIM-based abstain. The first two columns show the error rates when using the activation-based and the uncertainty-based abstain thresholds. The third column shows the error rates for the SSIM-based threshold. Here, we abstain from a prediction when the feature activation heatmaps achieve an SSIM score larger than or equal to 0.9. The SSIM-based abstain is a helpful addition for several datasets, which particularly shows in the last column where all three abstain thresholds are combined, which considerably lowers the error rates for all the datasets.

### 5.5. Evaluation on Real-World Social Media Data

Resizing and compression operations are applied throughout the experiments to simulate real-world environments. To further increase the realism of the experiments, we additionally test our architecture on out-of-distribution data, which are composed of data from social media platforms. More specifically, we utilize the TrueFace dataset by Boato et al. [59]. The dataset is composed of real and synthetic images, generated by the styleGAN1, styleGAN2, and styleGAN3 architectures before and after uploading to Facebook, Twitter, Telegram, and Whatsapp. The dataset is split into training and test data. For our evaluation, we use 100 images from the test dataset, where the synthetic images are generated by the styleGAN1 architecture. The images in these evaluations are severely out of distribution: neither the pre-social real images, nor the styleGAN1 generated images, nor the processing artifacts from real-world platforms like Facebook, Telegram, Twitter, or Whatsapp were observed during the training.

**Figure 5:**
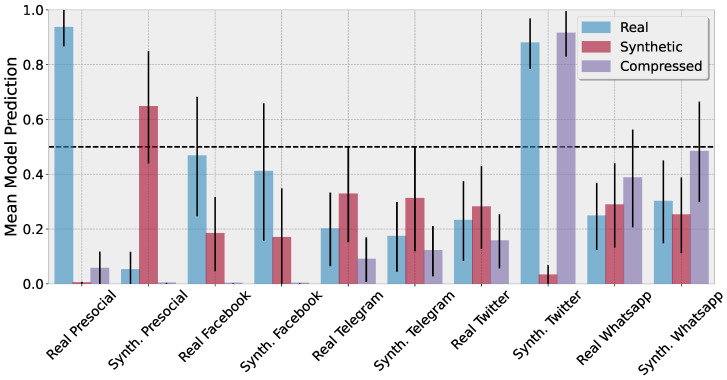
Mean model prediction on the TrueFace dataset [59] for real and synthetic data prior to and after uploading to Facebook, Telegram, Twitter, and Whatsapp. Synthetic images were generated by the StyleGAN architecture and for each evaluation we used 100 images from the test set. While images prior to the respective platform upload are on average correctly classified, our model shows highly increased uncertainty and abstains from predictions, rendering the post-social predictions unreliable. One notable exception includes synthetic images uploaded to Twitter, which are falsely classified as real images with high confidence. However, these false predictions can be detected by our SSIM-based threshold as these highly overlap with compression artifacts.

Figure 5 shows the mean predictions of our proposed architecture and the associated uncertainties as error bars. Our model shows high performance and confidence in its prediction on the real pre-social images. Synthetic pre-social images lead to higher uncertainty but can still be reasonably well detected. On the post-social images, our model shows, for the real and synthetic images, decreased class activation and highly increased uncertainty for almost every platform. One notable exception includes the synthetic images after uploading to Twitter. Here, our model wrongly classifies these as real with high confidence. However, at the same time, we can observe a high activation for the compressed class.

Table 5 shows the possibility to detect such unreliable false predictions. In fact, the false predictions on the out-of-distribution data can be reliably detected. The results in Table 5 show our model’s error rate in dependence of the abstain threshold on the TrueFace data. By using the combined approach, we are able to significantly reduce the error rate on the out-of-distribution data. This is especially shown for the synthetic images uploaded to Twitter. An assessment of the compression similarity (cf. Section 5.4) greatly reduces the initial error rate from 0.9 to 0.14 since the predictions for class “real” are rooted in confusion between the styleGAN and compression artifacts.

### 5.6. Ablation Study: Accuracy vs. Abstain Tradeoff by Uncertainty Thresholding

The uncertainty threshold $\sigma_{\mathrm{abstain}}$ has a major impact on reducing the error rate, as shown in the previous section. It also determines which predictions are deemed unreliable, which leads to abstaining from the predictions. In this section, we report the impact of the choice of $\sigma_{\mathrm{abstain}}$ on the error rate and abstain rate.

**Figure 6:**
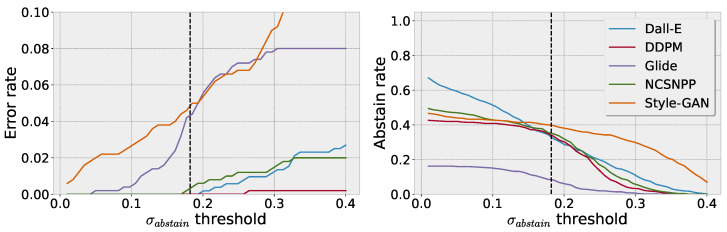
(**Left**) error rate as a function of the uncertainty threshold $\sigma_{\mathrm{abstain}}$. (**Right**) abstain rate as a function of the uncertainty threshold $\sigma_{\mathrm{abstain}}$. Choosing a more conservative $\sigma_{\mathrm{abstain}}$, the error rate is significantly reduced. However, the abstain rate on the other hand is increasing as more predictions are marked as unreliable. In both figures, the dotted line shows the $\sigma_{\mathrm{abstain}}$ threshold as chosen for our previous evaluations. For most cases, a threshold of twice the in-distribution model uncertainty shows a reasonable tradeoff between the error rate and abstain rate.

Figure 6 shows the tradeoff between the error rate and abstain rate of the BNN based on the chosen $\sigma_{\mathrm{abstain}}$. The left plot shows the error rate in dependency of the choice in threshold, where only predictions with an uncertainty smaller than $\sigma_{\mathrm{abstain}}$ are considered reliable. Here, lower thresholds substantially decrease the error rate. On the other hand, lower thresholds simultaneously increase the abstain rate, as indicated in the right plot of Figure 6.

The dotted black line shows the chosen uncertainty threshold we used throughout our previous experiments, which we specified as $\sigma_{\mathrm{abstain}}=2\cot\sigma_{\mathrm{in}}$, twice the in-distribution uncertainty. In our experiments, this choice yields a good tradeoff between a reduction in the error rate and an increase in the abstain rate.

### 5.7. Ablation Study: Effectiveness of the Noise Contrastive Estimation

We show the effects of the noise contrastive prior on the uncertainty estimates of the BNN in an ablation study. The BNN is trained with and without the NCP. Both models are trained with the same protocol, as specified in Section 4.

**Figure 7:**
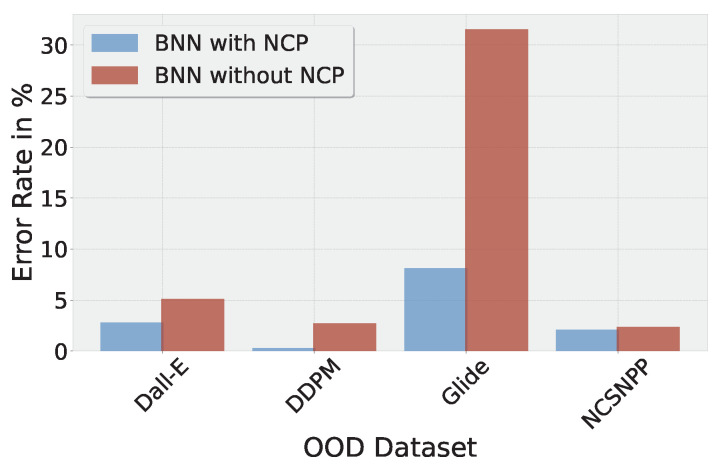
Comparison of BNN error rate on different OOD datasets with and without noise contrastive estimation (lower is better).

In Figure 7, we compare the error rate of the BNN without the NCP (“BNN-noNCP”) with the proposed BNN. The BNN without the NCP shows a higher error rate on all four out-of-distribution datasets. The difference in error rates is particularly large for the Glide images.

Table 6 further underlines these results. It shows the abstain rate of the traditional CNN, the BNN without the NCP, and the full BNN. The CNN exhibits the lowest abstain rates since most of the decisions are based on very large activations and hence high confidence. BNN-noNCP shows higher uncertainty on the out-of-distribution datasets, which is reflected by the increasing abstain rates. This behavior is amplified by the proposed BNN with the NCP, which exhibits the highest abstain rates regarding the data it fails to generalize to, thereby avoiding confident false decisions.

Table 7 confirms this result by showing the proportion of confident false decisions. In this case, we classify a decision as confident if the class prediction is ≥0.9. While the traditional CNN approach shows a significant amount of confident false decisions on the out-of-distribution data, the BNN without the NCP halves the proportion of false decisions, and the BNN with the NCP again halves the proportion of false decisions.

## 6. Conclusions

In this work, we investigate the challenge of reliably identifying the synthetic images produced by GAN models and diffusion models, with a strong emphasis on out-of-distribution data. We propose a Bayesian Neural Network for the detection of out-of-distribution data that cannot be reliably classified. The uncertainties of the BNN are further enhanced in the training with noise contrastive priors. Our experiments show that the BNN detects synthetic images comparably well to other state-of-the-art detectors, but it comes with the added benefit of the uncertainty measure.

We investigate three specific approaches to effectively convert the BNN outputs into a criterion for abstaining from uncertain decisions: by thresholding on the class activations, on the uncertainty, or on the structural similarity of the Grad-CAM features. All three criteria are effective in reducing the error rate, and a combination of these three criteria even further reduces the error rate.

We hope that these findings will create new opportunities for robust and reliable synthetic image detection on images from unknown sources. 

## Figures and Tables

**Table 1:** In-distribution evaluation of the BNN model.

Class	Precision	Recall	F1-Score	AU-ROC Score
Real	0.97	0.95	0.96	0.990
Synthetic	0.97	0.96	0.96	0.990
Compressed	0.99	0.98	0.99	0.999

**Table 2:** Comparison of state-of-the-art synthetic image detectors on uncompressed images. Row-wise best results are shown in bold.

Acc/AUC%	Spec [52]	PatchFor [53]	Wang [15]	Grag [19]	Ours	Ours (Activation Abstain)	Ours (Uncertain Abstain)
ProGAN	83.5/99.2	64.9/97.6	99.9/100	**99.9/100**	95.4/99.1	98.8/99.7	99.5/99.9
StyleGAN2	65.3/72.0	50.2/88.3	74.0/97.3	**98.1/99.9**	77.9/90.8	78.5/91.8	80.4/92.0
StyleGAN3	33.8/4.4	50.0/91.8	58.3/95.1	**91.2/99.5**	50.0/66.8	50.9/68.6	52.2/73.0
BigGAN	73.3/80.5	52.5/85.7	66.3/94.4	**95.6/99.1**	78.0/90.0	78.8/90.2	81.7/89.0
Dall-E mini	80.1/88.1	51.5/82.2	51.7/60.6	70.4/**95.6**	82.8/93.2	86.3/94.3	**88.6**/94.1
Dall-E 2	82.1/93.3	50.0/52.5	50.3/85.8	**51.9/94.9**	50.6/51.0	50.8/54.1	51.0/55.0
Stable Diffusion	66.8/74.7	50.8/85.0	50.9/65.9	62.1/**92.9**	74.4/88.6	74.5/87.5	**75.6**/89.1
Latent Diffusion	72.1/78.5	51.8/84.3	51.0/62.5	58.2/**91.5**	70.6/79.6	72.3/85.0	**72.6**/86.0
Taming Tran.	79.6/86.6	50.5/69.4	51.2/66.5	**73.5/96.6**	53.9/64.4	59.9/75.8	62.3/78.4
AVG	70.5/75.2	51.9/83.2	59.5/78.6	**75.8/92.8**	70.4/80.4	72.3/83.0	73.8/84.1

**Table 3:** Comparison of state-of-the-art synthetic image detectors on resized and compressed images. Row-wise best results are shown in bold.

Acc/AUC%	Spec [52]	PatchFor [53]	Wang [15]	Grag [19]	Ours	Ours (Activation Abstain)	Ours (Uncertain Abstain)
ProGAN	49.7/48.5	50.4/65.3	99.7/100	**99.9/100**	90.2/95.6	91.5/97.4	95.8/98.2
StyleGAN2	51.8/50.5	50.8/73.6	54.8/85.0	**63.3/94.8**	57.1/54.0	60.9/61.8	62.3/66.2
StyleGAN3	52.9/51.9	50.2/76.7	54.3/86.4	**58.3/94.4**	49.2/65.0	50.6/61.2	50.0/66.2
BigGAN	52.1/52.2	50.5/58.8	55.4/85.9	**79.0/99.1**	66.6/77.8	66.7/80.7	68.1/85.3
Dall-E mini	59.1/61.9	50.1/68.7	51.1/66.2	62.3/**95.4**	77.1/86.5	78.5/90.0	**80.6**/87.3
Dall-E 2	62.0/65.0	49.7/58.4	50.0/44.8	50.0/**64.4**	50.4/52.0	50.4/53.0	**51.3**/54.6
Stable Diffusion	46.5/44.5	51.1/77.2	50.7/72.9	58.1/**93.7**	74.4/88.6	74.5/87.6	**75.6**/89.2
Latent Diffusion	47.9/46.3	50.6/65.2	50.7/69.1	52.4/**89.4**	70.6/79.7	72.5/84.0	**72.6**/85.0
Taming Tran.	49.0/49.1	50.0/64.1	50.5/71.0	**56.2/94.3**	51.6/49.4	51.9/52.0	53.0/54.6
AVG	52.7/52.7	50.4/69.2	55.8/75.0	61.5/**90.8**	65.2/72.1	66.4/74.2	**67.7**/76.3

**Table 4:** BNN error rate (lower is better) in dependence of the abstain threshold for various in- and out-of-distribution data. Row-wise best results are shown in bold.

Dataset	Activation-Based	Uncertainty-Based	SSIM-Based	Combined
COCO	0.006	0.002	0.006	**0.002**
Stable Diffusion	0.004	0.000	0.004	**0.000**
Dall-E 2	0.027	0.000	0.023	**0.000**
DDPM	0.002	0.000	0.000	**0.000**
Glide	0.080	0.044	0.034	**0.016**
NCSNPP	0.020	0.004	0.016	**0.002**
StyleGAN2	0.264	0.046	0.136	**0.040**
LSUN	0.226	0.050	0.216	**0.048**
UFDD	0.064	0.016	0.030	**0.008**

**Table 5:** BNN error rate (lower is better) in dependence of the abstain threshold on the TrueFace test dataset. Row-wise best results are shown in bold.

Dataset	Activation-Based	Uncertainty-Based	SSIM-Based	Combined
Real Presocial	0.000	0.000	0.000	**0.000**
Synth. Presocial	0.010	0.000	0.010	**0.000**
Real Facebook	0.500	0.120	0.460	**0.120**
Real Telegram	0.140	0.040	0.120	**0.040**
Real Twitter	0.150	0.020	0.120	**0.020**
Real Whatsapp	0.160	0.060	0.100	**0.030**
Synth. Facebook	0.340	0.080	0.330	**0.080**
Synth. Telegram	0.070	0.010	0.060	**0.010**
Synth. Twitter	0.900	0.740	0.160	**0.140**
Synth. Whatsapp	0.250	0.070	0.120	**0.050**

**Table 6:** Comparison of the abstain rates of CNN, BNN, and NCP-BNN.

Model	COCO	Stable Diffusion	Dall-E	DDPM	Glide	NCSNPP
CNN	0.01	0.00	0.17	0.01	0.01	0.00
BNN-noNCP	0.03	0.00	0.16	0.14	0.27	0.15
BNN (proposed)	0.05	0.03	0.29	0.57	0.84	0.49

**Table 7:** Comparison of confident false decisions of CNN, BNN, and NCP-BNN (lower is better). The best results are shown in bold.

Model	In-Distribution	Out-of-Distribution
CNN	0.04	0.34
BNN-noNCP	0.02	0.15
BNN (proposed)	**0.01**	**0.07**

## Data Availability

The original data presented in the study are openly available at COCO—https://cocodataset.org/#home (accessed on 17 April 2023); UCID—https://faui1-files.cs.fau.de/public/mmsec/datasets/ucid.zip (accessed on 26 April 2023); ImageNet—https://image-net.org/ (accessed on 4 May 2023); GAN and DM Images—https://www.grip.unina.it (accessed on 5 August 2023); ProGAN—https://github.com/PeterWang512/CNNDetection (accessed on 18 September 2023); TrueFace—https://bit.ly/3bAEH75 (accessed on 11 April 2024). The data generated by the authors that support the findings will be available at https://faui1-files.cs.fau.de/public/mmsec/datasets/ROODROSI.zip from the date of publication.

## Abbreviations

The following abbreviations are used in this manuscript:

CNN	Convolutional Neural Network
GAN	Generative Adversarial Network
DM	Diffusion Model
OOD	Out-of-distribution
BNN	Bayesian Neural Network
NCE	Noise Contrastive Estimation
NCP	Noise Contrastive Prior
SVI	Stochastic Variational Inference
